# Heritable induced resistance in *Arabidopsis thaliana* : Tips and tools to improve effect size and reproducibility

**DOI:** 10.1002/pld3.523

**Published:** 2023-08-25

**Authors:** L. Furci, D. Pascual‐Pardo, L. Tirot, P. Zhang, A. Hannan Parker, J. Ton

**Affiliations:** ^1^ Plants, Photosynthesis and Soil, School of Biosciences, Institute for Sustainable Food The University of Sheffield Sheffield UK; ^2^ Plant Epigenetics Unit Okinawa Institute of Science and Technology Onna Okinawa Japan

**Keywords:** Arabidopsis, biotic stress, epigenetics, heritable induced resistance, *Pseudomonas syringae* pv. *tomato* DC3000

## Abstract

Over a decade ago, three independent studies reported that pathogen‐ and herbivore‐exposed *Arabidopsis thaliana* produces primed progeny with increased resistance. Since then, heritable induced resistance (h‐IR) has been reported across numerous plant‐biotic interactions, revealing a regulatory function of DNA (de)methylation dynamics. However, the identity of the epi‐alleles controlling h‐IR and the mechanisms by which they prime defense genes remain unknown, while the evolutionary significance of the response requires confirmation. Progress has been hampered by the relatively high variability, low effect size, and sometimes poor reproducibility of h‐IR, as is exemplified by a recent study that failed to reproduce h‐IR in *A. thaliana* by *Pseudomonas syringae* pv. *tomato* (*Pst*). This study aimed to improve h‐IR effect size and reproducibility in the *A. thaliana* –*Pst* interaction. We show that recurrent *Pst* inoculations of seedlings result in stronger h‐IR than repeated inoculations of older plants and that disease‐related growth repression in the parents is a reliable marker for h‐IR effect size in F1 progeny. Furthermore, RT‐qPCR‐based expression profiling of genes controlling DNA methylation maintenance revealed that the elicitation of strong h‐IR upon seedling inoculations is marked by reduced expression of the chromatin remodeler DECREASE IN DNA METHYLATION 1 (*DDM1*) gene, which is maintained in the apical meristem and transmitted to F1 progeny. Two additional genes, *MET1* and CHROMOMETHYLASE3 (*CMT3*), displayed similar transcriptional repression in progeny from seedling‐inoculated plants. Thus, reduced expression of *DDM1*, *MET1*, and *CMT3* can serve as a marker of robust h‐IR in F1 progeny. Our report offers valuable information and markers to improve the effect size and reproducibility of h‐IR in the *A. thaliana* –*Pst* model interaction.

## 1 INTRODUCTION

Plants increase their defensive capacity after recovery from pests or diseases. This induced resistance (IR) improves their performance against future attacks and is typically based on a combination of prolonged upregulation of inducible defenses and priming of inducible defenses (Wilkinson et al., 2019). The classic example is systemic acquired resistance (SAR), which develops after local pathogen attack and involves regulation by the NPR1 protein and the signaling metabolites salicylic acid (SA) and N‐hydroxy‐pipecolic acid (Zeier, 2021). Systemic IR can also be triggered by beneficial soil microbes (Pieterse et al., 2014), herbivory (Kloth & Dicke, 2022; Trapet et al., 2020), or chemical IR agents such as beta‐aminobutyric acid (BABA), benzothiadiazole, and (*R*)‐beta‐homoserine (Tao et al., 2022; Yassin et al., 2021). While the mechanisms controlling the onset and expression of IR have been studied intensely, comparably little is known about the mechanisms controlling the maintenance of IR. Remarkably, one of the first systematic studies of IR in tobacco reported persistence for 42 days in newly formed leaves (Bozarth & Ross, 1964), indicating a self‐perpetuating signal that is transmitted and maintained through cell division. Only decades later, researchers began to examine the long‐term maintenance of IR. In *Arabidopsis thaliana*, Luna et al. (2014) showed that priming of SA‐inducible defense genes and IR against biotrophic pathogens persists for 4 weeks after seedling treatment with BABA (Luna et al., 2014), while Wilkinson et al. (2023) reported that priming of jasmonic acid (JA)‐dependent defense genes and IR against herbivory is still present 3 weeks after transient JA stress (Wilkinson et al., 2023). Both studies also revealed regulatory functions of histone modifications and DNA methylation, supporting a growing body of evidence for epigenetic regulation of IR (Hannan Parker et al., 2022).

Histone modifications to the N‐terminal tail of histone proteins control chromatin density and transcription, which can be transmitted through cell division (Zhao et al., 2019). The formation of open chromatin occurs during IR at primed promoters of defense genes (Baum et al., 2019; Jaskiewicz et al., 2011), offering a plausible mechanism for the increased transcriptional capacity of these genes. Chromatin density in non‐coding regions of the genome, such as repetitive intergenic sequences and/or transposons, is often causally linked with DNA methylation, which recruits chromatin re‐modelers to repress transcription of potentially deleterious transposons. DNA methylation in plants, which is established and maintaned via different interdependent pathways, predominantly occurs at cytosines in different sequence contexts (CG, CHG and CHH, H indicates A C or T) (Zhang et al., 2018). IR‐eliciting stresses have been shown to induce dynamic changes in DNA methylation (Hannan Parker et al., 2022). Moreover, unlike animals, plants only partially reset acquired changes in DNA methylation during reproduction (Bouyer et al., 2017), providing an opportunity to transmit epigenetically acquired traits to the next generation. Indeed, artificially induced DNA demethylation can remain stable for 16 generations in epigenetic recombinant inbred lines (epiRILs) of *A. thaliana* (Cortijo et al., 2014). Moreover, some of these epialleles induce resistance against biotrophic pathogens via priming of defense genes (Furci et al., 2019). Because biotic stress has been linked to DNA demethylation (Hannan Parker et al., 2022), stress‐inducible epialleles provide a pathway by which IR can be transmitted to following generations.

Heritable IR (h‐IR) was first reported by Roberts (1983), who demonstrated that progeny from tobacco mosaic virus‐infected tobacco developed smaller lesions upon challenge inoculation with the same virus (Roberts, 1983). Over the following decades, various studies reported phenotypic changes in progeny from stress‐exposed plants (e.g., Molinier et al., 2006; Holeski, 2007), but it wasn't until the early 2010s that independent groups showed that exposure of plants to pathogens or herbivores can lead to heritable priming and IR in their progeny (Kathiria et al., 2010; Luna et al., 2012; Rasmann et al., 2012; Slaughter et al., 2012). Since then, h‐IR against biotic stress has been reported across a range of plant species (Table 1). In *A. thaliana*, mutants in the establishment and maintenance of DNA methylation mimic the primed defense state of h‐IR (López Sánchez et al., 2016; Luna & Ton, 2012), while mutations of the DNA demethylase ROS1 reduce basal resistance and block h‐IR against pathogens (Halter et al., 2021; López Sánchez et al., 2016). Together, these results strongly indicate that the dynamic removal of DNA methylation followed by DNA re‐methylation is a critical factor in the establishment, transmission, and/or expression of h‐IR in *A. thaliana*.

**TABLE 1:** Published cases of heritable induced resistance (h‐IR) against pests and diseases.

Plant species	Induction^a^	Challenge^b^	Phenotype^c^	Stability^d^	Reference
*Nicotiana tabacum*	Tobacco mosaic virus (TMV)	TMV	Resistance	F1	(Roberts, 1983)
*N. tabacum*	TMV	TMV, *Pseudomonas syringae* pv. *tomato*, *Phytophtora nicotianae*	Resistance	F1	(Kathiria et al., 2010)
*Arabidopsis thaliana*	*P. syringae* pv. *tomato*	*P. syringae* pv. *tomato, Hyalopernospera arabidopsidis*	Resistance	F1–F2	(Luna et al., 2012)
		*Alternaria brassicicola*	Susceptibility	F1	
*A. thaliana*	*P. syringae AvrRpt2*, *β‐aminobutyric acid* (BABA)	*P. syringae* pv. *tomato*, *H. arabidopsidis*	Resistance	F1	(Slaughter et al., 2012)
*A. thaliana*	*P. syringae* pv. *tomato*	*H. arabidopsidis*	Resistance	F1	(Luna & Ton, 2012)
*A. thaliana*, *Solanum lycopersicum*	*Pieris rapae*, *Helicoverpa zea*, Jasmonic acid	*P. rapae*, *H. zea*	Resistance	F1–F2	(Rasmann et al., 2012)
*Triticum aestivum*	Benzothiadiazole (BTH)	*Rhynchosporium commune*	Resistance	F1	(Walters & Paterson, 2012)
*Brassica rapa*	Cauliflower mosaic virus (CaMV)	CaMV	Resistance	F1	(Kalischuk et al., 2015)
*Solanum tuberosus*	BABA	*Phytophthora infestans*	Resistance	F1	(Floryszak‐Wieczorek et al., 2015)
*Phaseolus lunatus*	*Gynandrobrotica guerreroensis*	*G. guerreroensis*	Resistance	F1	(Ballhorn et al., 2016)
*S. lycopersicum*	*Trichoderma atroviride*	*Meloidogyne javanica*	Resistance	F1	(Medeiros et al., 2017)
*A. thaliana*	*Tetranychus urticae*	*T. urticae*	Resistance	F1–F2	(Singh et al., 2017)
		*Myzus persicae*	Resistance	F1	
		*P. syringae* pv. *tomato*	Susceptibility	F1–F2	
*Phaseolus vulgaris*	BABA	*P. syringae* pv. *phaseolicola*	Resistance	F1	(Ramírez‐Carrasco et al., 2017)
*S. tuberosus*	BABA	*P. infestans*	Resistance	F1	(Meller et al., 2018)
*A. thaliana*	*P. syringae* pv. *tomato*	*H. arabidopsidis*	Resistance	F1–F3	(Stassen et al., 2018)
*S. tuberosus*	*BABA*	*P. infestans*	Resistance	F1	(Kuźnicki et al., 2019)
*Nicotiana attenuata*	*Manduca sexta*	*M. sexta*	Resistance	F1	(Kafle & Wurst, 2019)
		*Meloidogyne incognita*	Susceptibility		
*P. vulgaris*	*Rhizobium etli*	*P. syringae* pv. *phaseolicola*	Resistance	F1	(Díaz‐Valle et al., 2019)
*Castanea sativa* Miller	*Phytophthora cinnamomi*	*P. cinnamomi*	Resistance	F1	(Camisón et al., 2019)
*P. vulgaris*	BTH	*Xanthomonas axonopodis* pv. *phaseoli*	Resistance	F1	(Akköprü, 2020)
*Plantago lanceolata*	*Podosphaera plantaginis*	*P. plantaginis*	Resistance	F1	(Höckerstedt et al., 2021)
*Quercus ilex*	*P. cinnamomi*	*P. cinnamomi*	Resistance	F1	(Vivas et al., 2021)
*A. thaliana*	*P. syringae* pv. *tomato*	*P. syringae pv. tomato*, *H. arabidopsidis*	Resistance	F1–F2	(López Sánchez et al., 2021)
		*P. cucumerina*	Susceptibility	F1	
	*P. cucumerina*	*P. cucumerina*	Resistance	F1–F2	
		*H. arabidopsidis*	Susceptibility	F1	
*P. vulgaris*	2,6 dichloro‐isonicotinic acid	*P. syringae* pv. *phaseolicola*	Resistance	F1	(Martínez‐Aguilar et al., 2021)
*T. aestivum*	*Trichoderma asperellum*	*Bipolaris sorokiniana*	Resistance	F1	(Tiwari et al., 2022)
*Oryza sativa*	*Meloidogyne graminicola*	*M. graminicola*	Resistance	F1	(Meijer et al., 2023)

^a^ Biotic or chemical stimulus eliciting h‐IR in parental plants.

^b^ Effectiveness of the h‐IR response in progeny.

^c^ h‐IR phenotype in terms of resistance or susceptibility to the same and/or other stresses.

^d^ Generation in which h‐IR was still apparent following parental stress.

Despite mounting evidence for h‐IR across numerous plant‐biotic stress interactions (Table 1), it remains unknown which DNA demethylated loci drive the response and how these epialleles prime defense genes and induce resistance. This progress is hampered by a combination of factors. Apart from the highly quantitative nature of resistance‐inducing epialleles (Furci et al., 2019), DNA demethylated epialleles can prime defense genes via *trans*‐acting mechanisms (reviewed by Cooper & Ton, 2022), making it challenging to link stress‐inducible epialleles to primed defense genes. Another limitation is the variability and reproducibility of h‐IR. This is exemplified by a recent study reporting a series of unsuccessful attempts to reproduce h‐IR in the *A. thaliana*–*Pseudomonas syringae* DC3000 (*Pst*) interaction (Yun et al., 2022). Inspired by this report, the objective of this study was to improve the reproducibility and effect size of h‐IR for the *A. thaliana*–*Pst* interaction, and so facilitate future research on this epigenetic plant response. Here, we present new evidence that the intensity of parental disease stress is a crucial factor for h‐IR in F1 progeny. We furthermore show that *Pst* inoculation of seedlings leads to a stronger h‐IR response, which is marked by repressed transcription of genes controlling DNA methylation maintenance in infected leaves, meristematic tissue, and untreated F1 seedlings.

## 2 RESULTS

### 2.1 Recurrent *Pst* inoculations of seedlings results in stronger h‐IR than recurrent *Pst* inoculations of older plants

**FIGURE 1:**
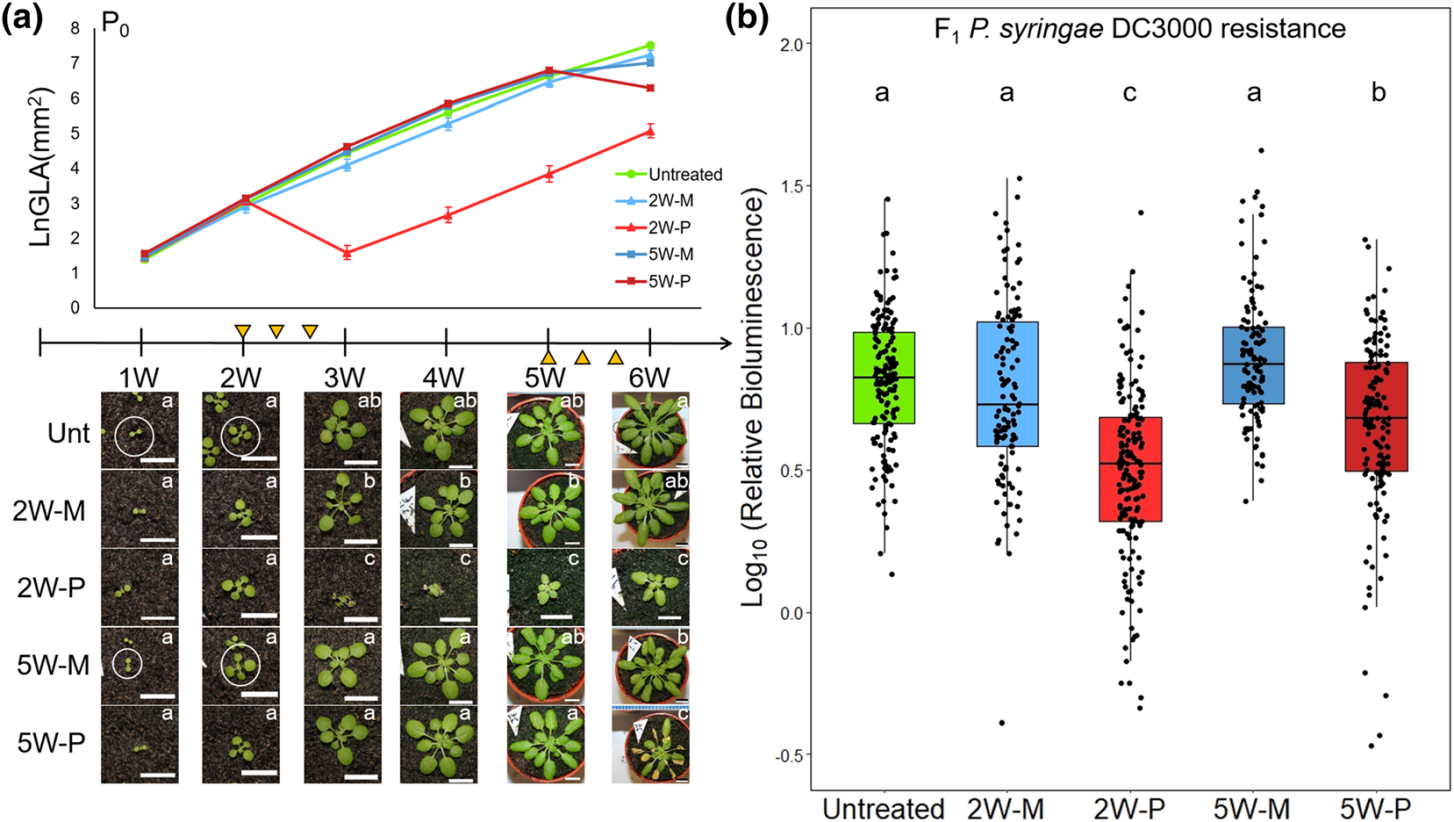
Effects of parental age at disease exposure on heritable induced resistance (h‐IR) in *Arabidopsis thaliana* against *Pseudomonas syringae* pv. tomato DC3000 (*Pst*). (a) Growth phenotypes of parental plants in response to *Pst* stress treatments. Shown are average values of Ln‐transformed green leaf area (GLA) of untreated plants (Unt) and plants after three successive mock (M) or Pst (P) inoculations. Plants were inoculated either as seedlings between 2 and 3 weeks (2W), or as older plants between 5 and 6 weeks (5W), as indicated by the yellow triangles. Photographs show representative phenotypes of the same plant over the time course of the experiment. Scale bar = 1 cm. Letters inside photographs indicate statistically significant differences between treatments at each time‐point analyzed (one‐way ANOVA followed by Tukey's post‐hoc test, alpha = .05, *n* = 6, ±standard error of the mean). (b) Colonization of *Pst::LUX* in leaves of F1 progeny from untreated, mock‐inoculated and *Pst*‐inoculated 2W and 5W plants. Shown are Log_10_‐transformed values of relative bioluminescence at 2 days after inoculation of 2‐week‐old F1 seedlings. Letters indicate statistically significant differences between treatments (Welch's ANOVA followed by Games–Howell post hoc test, α = .05, *n* > 110).

Yun et al. (2022) proposed that h‐IR by *Pst* in *A. thaliana* is caused by disease progression into the flower stalk, exposing F1 embryos to disease stress. To investigate this hypothesis, we performed an experiment in which 2‐ to 3‐week seedlings (2W) were repeatedly inoculated with *Pst* carrying the *luxCDABE* operon (*Pst::LUX*), allowing plants to recover from the disease before the onset of flowering at 6 weeks. A complementary set of plants were inoculated between 5 and 6 weeks (5W), allowing for *Pst* disease to progress into the inflorescence. Over the 1‐week period of successive *Pst*::*LUX* inoculations, plants were maintained at 100% relative humidity (RH) to promote disease. To control for stress by 100% RH, an untreated group was included that was not inoculated and maintained at ambient RH. Monitoring green leaf area (GLA) over the course of the experiment revealed statistically significant reductions in vegetative growth by *Pst::LUX*, which were more pronounced in 2W plants than 5W plants (Figure 1a). Six weeks after planting, all plants were moved to long‐day conditions to trigger flowering. At this point, 2W plants were free of disease symptoms, whereas 5W plants still showed symptoms (Figure 1a). To confirm that *Pst* is no longer present in the floral tissues of 2W plants, we performed PCR amplification of the bacterial Luciferase gene in DNA extracts from flower buds of 2W plants at 53 days after the final *Pst::LUX* inoculation and from 2W seedlings at 2 days after the final inoculation. In contrast to the PCR analysis of inoculated 2W seedlings, no bacterial DNA could be detected in the floral tissues of inoculated 2W plants, while PCR of plant DNA yielded a positive product in all tissues (Figure S1). Hence, the F1 embryos in the developing flower buds of 2W were not directly exposed to the pathogen.

To quantify h‐IR, 2‐week‐old F1 progeny were challenged with bioluminescent *Pst::LUX* and analyzed for bacterial colonization (Furci et al., 2021). Compared with untreated and mock‐treated controls, progeny from *Pst*‐inoculated 2W and 5W plants showed a statistically significant reduction in *Pst::LUX* colonization. Interestingly, this h‐IR was statistically stronger in F1 progeny from 2W plants compared with F1 progeny from 5WP plants (Figure 1b), showing that *Pst* inoculations of seedlings yield stronger h‐IR than similar treatments of older plants. Because 2W plants had recovered from *Pst* disease before flowering, it is unlikely that h‐IR is caused by disease exposure of F1 embryos in the flowers.

### 2.2 Disease‐related growth repression in the parents determines h‐IR effect size in the F1

**FIGURE 2:**
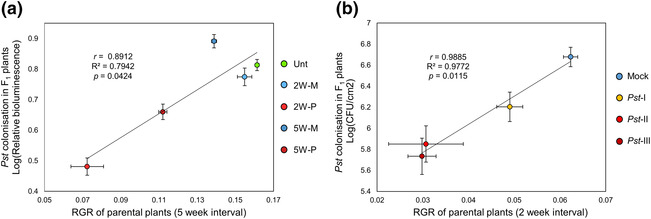
The relationship between parental disease stress and heritable induced resistance (h‐IR) effect size in F1 progeny. Correlation plots show *Pst* leaf colonization (Log_10_‐transformed values) in F1 progeny as a function of parental plant growth (relative growth rates; RGRs) under varying degrees of *Pst* disease stress. Shown are the results from two independent experiments. (a) Plot is based on data form the current study (Figure 1). (b) Plot is based on data from a previous h‐IR experiment by López Sánchez et al. (2021), in which 4.5‐week‐old plants were either mock‐inoculated (Mock), or *Pst*‐inoculated 2, 4, or 6 successive times (*Pst*‐I, *Pst*‐II, and *Pst*‐III, respectively). Inserts show the Pearson correlation (*r*), the coefficient of determination (*R* ^2^), and the statistical significance (*p*) of the regression. Error bars indicate standard error of the mean.

Because *Pst* stress in 2W plants was more severe than in 5W plants (Figure 1a), we hypothesized that the strength of h‐IR is proportional to the disease severity experienced by the parents. Indeed, relative growth rate (RGR) reduction in parental plants was positively correlated with weaker *Pst* colonization in F1 progenies (Figure 2a). To validate this outcome, we analyzed data from a previous h‐IR experiment in which 4.5‐week‐old parental plants had been exposed to increasing levels of *Pst* stress (López Sánchez et al., 2021). Although this experiment was conducted by different researchers in our laboratory using different growth conditions and methods to quantify *Pst* colonization, it showed a similar correlation between parental RGR and *Pst* colonization in F1 progeny (Figure 2b). Hence, h‐IR is only evident when parental disease stress is sufficiently severe to cause substantial reductions in growth (>25% RGR).

### 2.3 Severe parental disease stress induces prolonged repression of genes controlling DNA methylation

**FIGURE 3:**
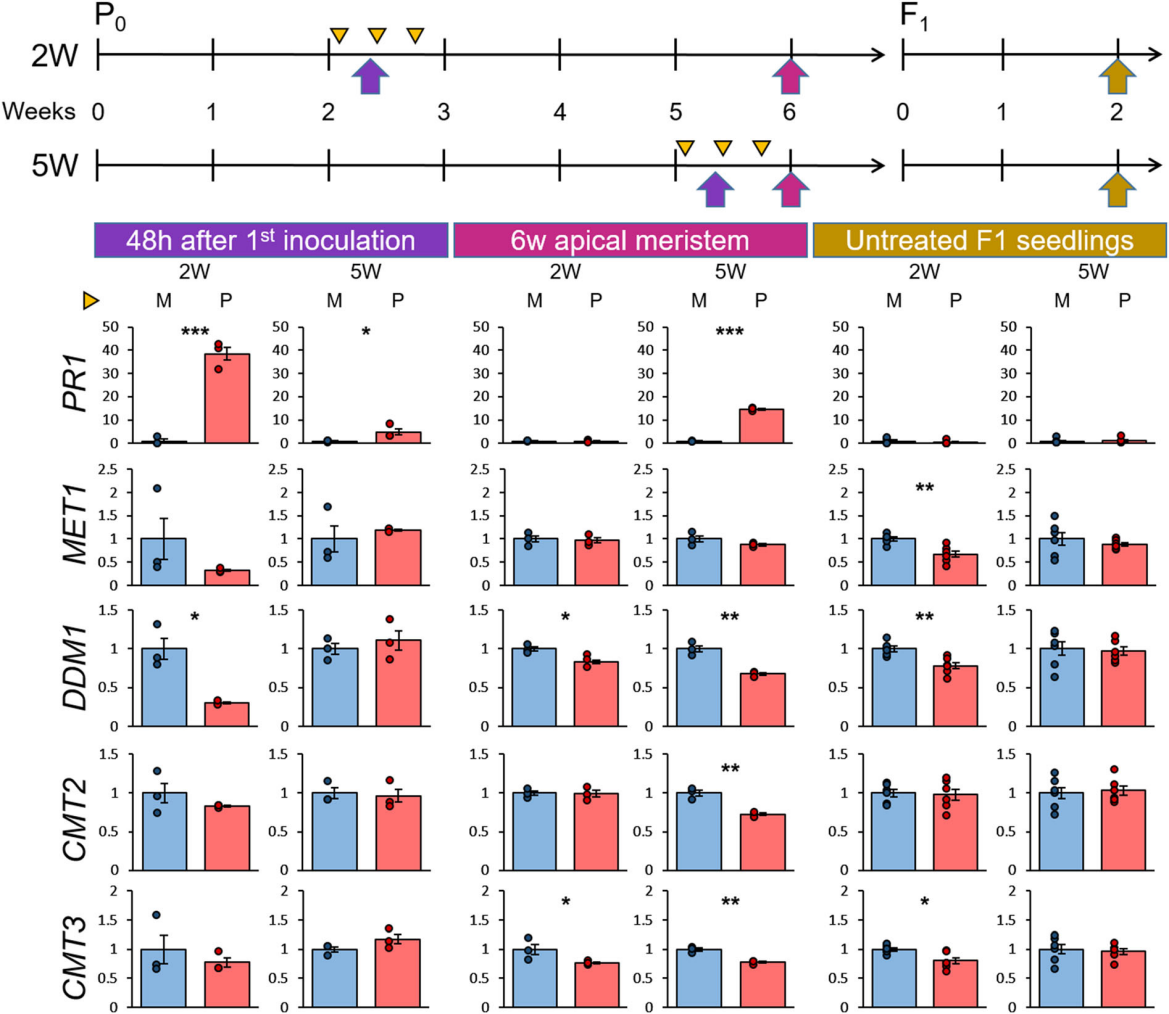
Expression profiles of the stress‐responsive *PR1* gene and the DNA methylation maintenance genes *MET1*, DECREASED IN DNA METHYLATION 1 (*DDM1*), CHROMOMETHYLASE2 (*CMT2*), and CHROMOMETHYLASE3 (*CMT3*) during the establishment and generational maintenance of heritable induced resistance (h‐IR). Shown are the mean relative expression values (*n* = 3–6; ±standard error of the mean) of *PR1*, *MET1*, *DDM1*, *CMT2*, and *CMT3* at different time‐points after mock inoculation (M; blue bars) or *Pst* inoculation (P; red bars) of parental plants. Mock and *Pst* inoculations are indicated in the experimental timelines at the top by yellow triangles; the time‐points of RNA sampling are indicated by colored arrows (purple: leaf tissue at 48 h after the first inoculation; magenta: apical meristem of 6‐week‐old plants; yellow: leaf tissues of 2‐week‐old F1 seedlings). Asterisks indicate statistically significant differences between parental treatments (Student's *t*‐tests; *p < .05; **p < .01; ***p < .001).

*Pst* disease in *A. thaliana* represses genes controlling DNA methylation (Yu et al., 2013). To investigate whether this transcriptional response is related to h‐IR, we profiled the expression of five key genes controlling DNA methylation maintenance in leaves at 48 h after primary *Pst* inoculation, in the apical meristem of 6‐week‐old plants, and in leaves of 2‐week‐old F1 seedlings (Figure 3). The SA‐inducible *PR1* gene was included to mark disease stress. At 48 h after *Pst* inoculation, *PR1* showed approximately a 40‐fold induction in 2W seedlings compared to only a 5‐fold induction in 5W plants, confirming that 2‐week‐old seedlings experience more severe stress from *Pst* than 5‐week‐old plants. The apical meristem of *Pst*‐inoculated 5W plants, which showed symptoms 2 days after the third *Pst* inoculation (Figure 1a), showed a 16‐fold induction of *PR1*. By contrast, *PR1* expression in the apical meristem of *Pst*‐inoculated 2W plants was reduced to basal levels 23 days after the third inoculation, confirming that these plants had fully recovered from disease stress before flowering (Figure 3). Expression of the *DECREASED IN DNA METHYLATION 1* (*DDM1*) gene, which controls DNA methylation maintenance at all sequence contexts (Zhang et al., 2018), showed statistically significant repression 48 h after *Pst* inoculation, which was more pronounced in 2W plants than in 5W plants. Twenty‐three days after the last *Pst* inoculation, the apical meristem of 6‐week‐old 2W plants still showed a statistically significant repression of the *DDM1* gene (Figure 3). Because these plants had fully recovered from disease stress by the time of sampling the meristematic tissues, it can be concluded that *Pst*‐induced *DDM1* repression is maintained throughout the vegetative life cycle of the plant. The apical meristem of 5W plants, which were still symptomatic at the time of sampling, also showed reduced expression of *DDM1*, in addition to reduced expression of *CHROMOMETHYLASE3* (*CMT3*) and *CHROMOMETHYLASE2* (*CMT2*), which maintain DDM1‐dependent non‐CG DNA methylation (Zhang et al., 2018). Analysis of 2‐week‐old F1 seedlings revealed that the repressed state of the *DDM1* gene in *Pst*‐inoculated 2W plants was still apparent in their F1 progeny. Similarly, *MET1* and *CMT3* expression showed statistically significant repression in F1 progeny from *Pst*‐inoculated 2W plants. By contrast, none of these regulatory genes showed differences in expression between F1 progenies from 5W plants (Figure 3). Thus, the relatively strong h‐IR response in progeny from *Pst*‐treated 2W plants is marked by transcriptional repression of genes controlling DNA methylation maintenance.

## 3 DISCUSSION

Our study shows that h‐IR in the *A. thaliana*–*Pst* interaction only occurs when parental plants experience severe disease stress that causes major growth reductions (Figures 1 and 2). This parent–offspring relationship was evident in independent experiments under different experimental conditions (Figure 2). Although *A. thaliana* develops visible water‐soaked lesions and chlorosis in the days following *Pst* inoculation, the h‐IR response in F1 progeny remains weak or absent if these symptoms are not accompanied by a substantial reduction in growth (Figures 1 and 2). We therefore recommend confirming sufficient growth repression by *Pst* before proceeding with the analysis of h‐IR‐related phenotypes in the next generation. In that regard, the relatively mild symptoms reported by Yun et al. (2022) would likely have been insufficient to cause h‐IR. Bacterial speck disease caused by *Pst* is non‐progressive in *A. thaliana*. Consequently, colonization of the pathogen typically peaks between 2 and 3 days after inoculation and dramatically declines by 5 days (Furci et al., 2021). Indeed, 6‐week‐old 2W plants no longer showed bacterial speck symptoms or elevated *PR1* gene expression at 23 days after the third *Pst* inoculation (Figures 1 and 3), and bacterial DNA was undetectable in the floral tissues that form the F1 embryos at 53 days after the final *Pst* inoculation (Figure S1), rendering disease progression into the inflorescence highly unlikely. Combined with our finding that recurrent *Pst* inoculations of 2W plants yield stronger h‐IR phenotypes than inoculations of 5W plants, our results do not support the hypothesis that h‐IR is caused by exposure of F1 embryos to *Pst* disease (Yun et al., 2022). A recent study reported h‐IR in progeny from plants treated with the root endoparasite *Meloidogyne graminicola* (Meijer et al., 2023), which is unable to colonize the stem or inflorescence, further discounting the hypothesis that h‐IR is caused by direct exposure of F1 embryos to disease.

*A. thaliana* develops SA‐dependent age‐related resistance (Wilson et al., 2017), which represses *Pst* disease. Accordingly, age‐related resistance can explain why progeny from *Pst*‐inoculated 5W plants showed relatively weak h‐IR. We therefore recommend performing *Pst* inoculations at earlier developmental stages, which induces more disease stress and thus improves h‐IR effect size (Figures 1 and 2a). We also recommend keeping plants at 100% RH throughout the *Pst* inoculations because *Pst* only causes disease in *A. thaliana* when kept at 100% RH for at least 2 days after inoculation (Xin et al., 2016). Stress caused by 100% RH should not be a confounding factor because F1 progeny from untreated plants and mock‐inoculated plants showed similar *Pst* susceptibility (Figure 1b). Apart from age‐related resistance and humidity, there are other factors that can negatively affect *Pst* disease. For instance, the light regime, soil type, and soil‐associated microbes can have a profound influence on *Pst* disease (Hassan et al., 2018; Roeber et al., 2021). It is also worth noting that our h‐IR assays in F1 plants are based on spray inoculations rather than leaf infiltrations, thereby assessing the contributions of both pre‐invasive and post‐invasive defenses. Finally, we recommend considering the ancestral history of the *A. thaliana* germline, particularly if seed stocks are maintained under greenhouse conditions that are not controlled for pests and diseases. As h‐IR can persist over multiple stress‐free generations (López Sánchez et al., 2021; Stassen et al., 2018), h‐IR from unaccounted ancestral stress by pests and/or diseases could mask h‐IR by *Pst*.

DDM1 is a chromatin remodeller that controls DNA methylation maintenance in all sequence contexts, targeting mostly repetitive DNA sequences in heterochromatic transposon‐rich regions (Zhang et al., 2018). Temporary loss of DDM1 activity induces demethylated epialleles that remain stable for at least 8–16 generations (Cortijo et al., 2014), some of which induce high levels of resistance (Furci et al., 2019). Our gene profiling revealed that severe disease in 2W seedlings causes repression of *DDM1*, which is maintained in the unstressed meristematic leaves and transmitted to F1 progeny (Figure 3). Two other genes involved in DNA methylation maintenance, *MET1* and *CMT3*, also showed statistically significant repression in F1 progeny from *Pst*‐treated 2W plants. This repression of DNA methylation machinery may contribute to reduced DNA methylation at transposon‐rich heterochromatic regions, which has been implicated in the control of heritable priming and h‐IR (Furci et al., 2019; López Sánchez et al., 2016; Luna & Ton, 2012). From a more practical perspective, repressed expression of *DDM1*, *MET1,* and *CMT3* can be used as a marker for robust h‐IR in the *A. thaliana*–*Pst* model system.

## 4 MATERIALS AND METHODS

### 4.1 Plant growth conditions and parental *Pst* inoculation

*A. thaliana* seeds (Col‐0) were stratified in water for 4 days in darkness at 4°C before sowing in a sand:M3 mixture (1:3). Plants were kept vegetative for 6 weeks under short‐day conditions (8.5 h light/15.5 h dark, 21°C, 60% RH, ~125 μmol s^−1^ m^−1^ light intensity) before transference to long‐day conditions (16 h light/8 h dark) to trigger flowering. After 2 and 5 weeks of vegetative growth, 2W and 5W plants were inoculated three times at a 2‐day intervals with bioluminescent *Pst* *::* *LUX* (Fan et al., 2008) at OD600 = .2 (for the first two inoculations) and OD600 = .3 (for the 3rd inoculation) in 10 mM MgSO_4_ supplemented with .01% v/v Silwet L‐77, or mock solution (10 mM MgSO_4_ + .01% v/v Silwet L‐77). Inoculated plants were kept for 2 days at 100% RH to promote disease, while untreated plants were kept at 60% RH throughout. To avoid sudden changes in RH during infection, digital photos were taken between inoculations after 2 days. *Pst* *::* *LUX* bacteria were cultured in a shaking incubator (Grant‐Bio; ES‐20) O/N at 28°C from 1 mL of frozen glycerol stocks in King's medium B, containing 50 μg/mL Rifampicin and Kanamycin. *Pst* *::* *LUX* cultures were centrifuged at 800*g* for 3 min., after which pellets were washed and re‐suspended in 10 mM MgSO4 to final density. Six plants per treatment were allowed to set seed for quantification of h‐IR.

### 4.2 Quantification of growth

Parental growth was captured by high‐resolution digital photography (Canon, 500D 15MP). Green pixels corresponding to GLA were selected by Adobe Photoshop 6.0. using a combination of “magic wand” and “lasso” tools and converted into mm^2^. For each plant, RGR was calculated over a 5‐week interval using the below formula (GLA2 = GLA at 6 weeks and GLA1 = GLA at 1 week; *t2* = 42 days and *t1* = 7 days):

$$\mathrm{RGR}=\frac{\left(\ln\;\mathrm{GLA}2-\ln\;\mathrm{GLA}1\right)}{\left(t2-t1\right)}$$

### 4.3 Quantification of h‐IR in F_1_ progeny

Two‐week‐old seedlings from pooled F1 progeny of four parental plants per treatment were challenged by spray‐inoculation with *Pst* *::* *LUX* in 10 mM MgSO_4_ supplemented with .01% v/v Silwet L‐77 (OD600 = .2) and kept at 100% RH for 2 days. Bacterial bioluminescence was captured by a G:BOX gel doc (Syngene). Relative bioluminescence in leaves was quantified as a function of pixel brightness (Furci et al., 2021).

### 4.4 RT‐qPCR assays

Biological replicates (*n* = 3–6) were collected at the time‐points indicated, each consisting of 6–12 leaves (expanded leaves or meristematic leaves) from three different plants per sample. Samples were snap‐frozen and pulverized in N_2_ (l), using a tissue lyser (QIAGEN TissueLyser) and steel beads. Total RNA was extracted using a guanidinium thiocyanate‐phenol‐chloroform protocol, as described previously (Furci et al., 2019). RNA extracts were treated with DNaseI using the RQ1 RNase‐Free DNase kit (Promega, M6101). First‐strand cDNA synthesis was based on 1 μg total RNA, using SuperScript III Reverse Transcriptase (Invitrogen, 18080093) according to the supplier's instructions. The qPCR reactions were performed with a Rotor‐Gene Q real‐time PCR cycler (Qiagen) using the Rotor‐Gene SYBR Green PCR Kit (Qiagen). Relative gene expression was calculated with correction for amplification efficiency as described previously (Furci et al., 2019). Gene expression was normalized to the mean expression values of two stably expressed genes (At5G25760 and At2G28390). Primer sequences are listed in Table S1.

### 4.5 PCR detection of *Pst::LUX*

PCR end‐point analysis to detect bacterial DNA (Luciferase) was performed in seedlings at 2 days after the final *Pst::LUX* inoculation (each replicate consisting of pooled shoots from six seedlings) and flower tissues at 53 days after the final *Pst::LUX* inoculation (each replicate consisting of ~20 pooled flower buds from 2 plants). DNA was extracted with the DNeasy Plant Kit (Qiagen; #69106). PCR was performed using primers against the luciferase gene from the transgenic *luxCDABE* operon and a plant gene (AtG28390; positive control). Primers are listed in Table S1. Reactions were performed with a Prime thermocycler (Techne) using a three‐step PCR program (30 cycles: denaturation at 95°C for 30 s, primer annealing at 58°C for 30 s, and primer extension at 68°C for 60 s) and Tag polymerase from NEB (#M0273L).

### 4.6 Statistical analyses

Differences in GLA between parental treatments were analyzed by ANOVA and Tukey HSD tests after verification of normal distributions and homoscedasticity using Levene's tests (SPSS 27). The statistical analysis of h‐IR in F1 progeny was performed by Welch's ANOVA followed by a Games–Howell post‐hoc test (SPSS 27). Differences in relative gene expression were analyzed by unpaired 2‐tailed Student's *t*‐tests. Pearson's correlation and linear regression were performed on treatment‐averaged RGR and *Pst* colonization values using R (v 4..4).

## AUTHOR CONTRIBUTIONS

L. Furci and J. Ton designed and conceived experiments, L. Furci, D. Pascual‐Pardo, L. Tirot, and P. Zhang conducted experiments, and L. Furci, J. Ton, and A. Hannan Parker analyzed the data. L. Furci and J. Ton wrote the manuscript text, with revisions by L. Tirot and A. Hannan Parker. J. Ton provided funding for the research.

## CONFLICT OF INTEREST STATEMENT

The Authors did not report any conflict of interest.

## PEER REVIEW

The peer review history for this article is available in the Supporting Information for this article.

## DATA AVAILABILITY STATEMENT

All biological material and raw data will be available upon request to the corresponding authors.

## Supporting information


**Table S1** Supporting Information

**Figure S1:** PCR detection of bacterial DNA (luciferase gene from *Pst::LUX*; left) and plant DNA (At2g28390 gene from Arabidopsis; right). Shown are PCR reactions using DNA from seedlings at 2 days after the third *Pst::LUX* inoculation (seedlings) and from flower tissues at 53 days after the third *Pst::LUX* inoculation (flowers). PCR reactions were performed on DNA extracts from three biologically samples per treatment. Sample Col‐0 represents DNA from uninfected plants.
