# Supplementary material for: Heritable induced resistance in Arabidopsis thaliana : Tips and tools to improve effect size and reproducibility
Source: Plant Direct. 2023 Aug 25;7(8):e523. doi: 10.1002/pld3.523 (PMC10457550; doi:10.1002/pld3.523)
Supplement: Supplementary file 1 — Table S1 Supporting Information [file PLD3-7-e523-s002.docx]

**Supplementary Table 1:**

| **RT-qPCR primer list** | | | |
| --- | --- | --- | --- |
| Gene Name | TAIR ID | Forward primer (5'->3' + strand) | Reverse primer (5'->3' - strand) |
| *UBC21* | At5G25760 | TCTCTCTCTCTCTCTCTCGCTCTC | TGATGCCTGCATCTCTAATTTCCC |
| *MON1* | At2G28390 | CAAGGCAGGAAATCACCAGGTTG | CTGTACAGCTGATGCAGACCAG |
| *PR1* | At2G14610 | ACACGTGCAATGGAGTTTGTGG | TTGGCACATCCGAGTCTCACTG |
| *MET1* | At5G49160 | CCAGTAGATTTCGCTTCTCAACGG | TTCGCTTCGCAGCTTTAGCC |
| *DDM1* | At5G66750 | TGGCCAAGGGCAGTTTCATCAAG | AACGCCAGTATGTCCTCTTCCTC |
| *CMT2* | At4G19020 | GCTCGTCTTTGGTGGGATGAAAC | TCAGGATGCAATAACGCCTGAC |
| *CMT3* | At1G69770 | TGTCGATGGGAAATCATGCAAACC | TTCTGCCCGTGTGACAACAG |
| **PCR primer list** | | | |
| Gene Name | TAIR ID/operon ID | Forward primer (5'->3' + strand) | Reverse primer (5'->3' - strand) |
| *MON1* | At2G28390 | AACTCTATGCAGCATTTGATCCACT | TGATTGCATATCTTTATCGCCATC |
| Luciferase | *LuxAB* | GCCAGAAGAAAACAGCCCA | TTCGGCGTTCATTGACTGTC |
