# Supplementary figures and images for: Heritable induced resistance in Arabidopsis thaliana : Tips and tools to improve effect size and reproducibility
Source: Plant Direct. 2023 Aug 25;7(8):e523. doi: 10.1002/pld3.523 (PMC10457550; doi:10.1002/pld3.523)

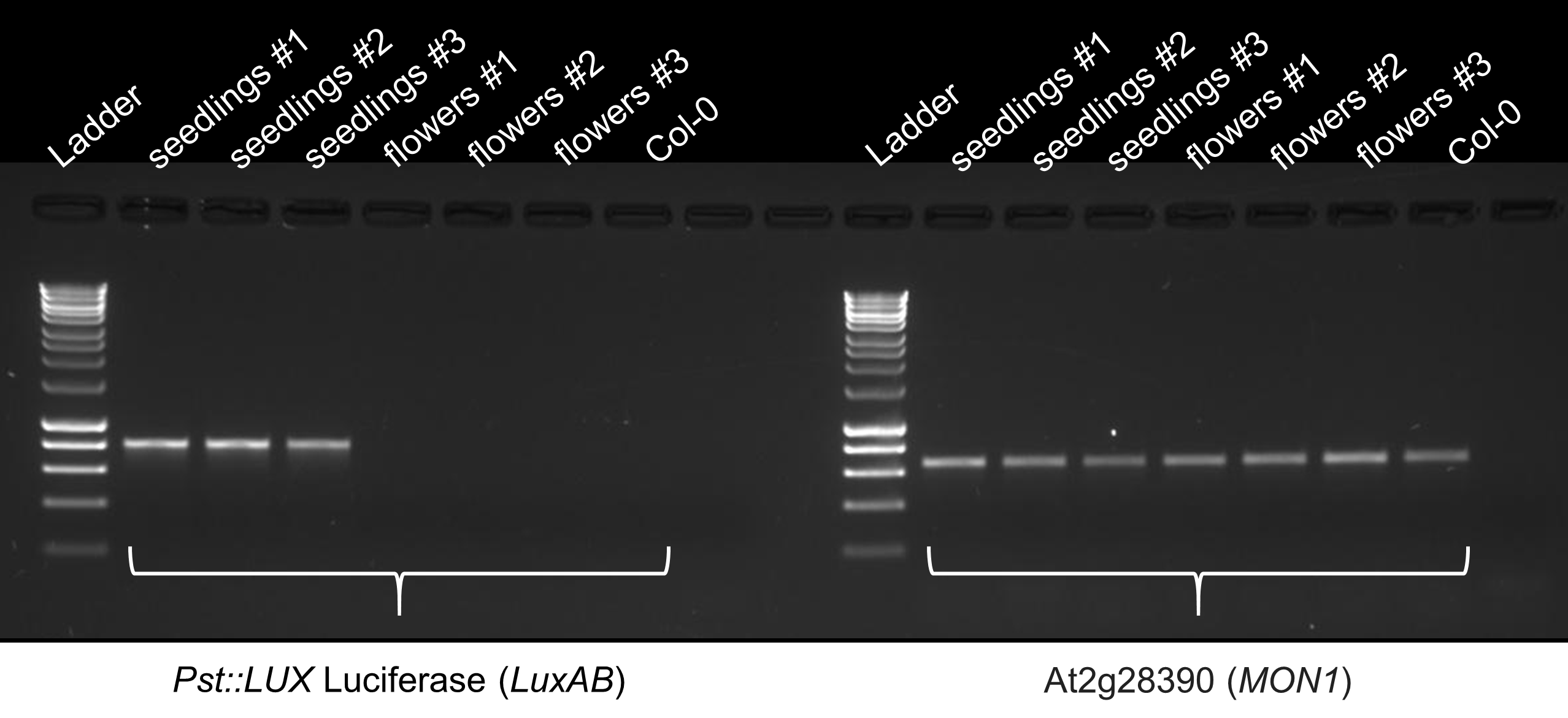

Supplement: Supplementary file 2 — Figure S1: PCR detection of bacterial DNA (luciferase gene from Pst::LUX; left) and plant DNA (At2g28390 gene from Arabidopsis; right). Shown are PCR reactions using DNA from seedlings at 2 days after the third Pst::LUX inoculation (seedlings) and from flower tissues at 53 days after the third Pst::LUX inoculation (flowers). PCR reactions were performed on DNA extracts from three biologically samples per treatment. Sample Col‐0 represents DNA from uninfected plants. [file PLD3-7-e523-s001.tif]
